# Comparison of different behaviour management techniques while administering injectable la with minimum pain perception and anxiety in children

**DOI:** 10.4317/jced.61736

**Published:** 2024-08-01

**Authors:** Ansari Heena, Manvi Malik, Prabhav Chauhan, Prachi Arora, Kopal Singh, Mudang Moniya

**Affiliations:** 1 MDS (P.G. 3rd Year Student), Department of Pediatric and Preventive Dentistry, Shree Bankey Bihari Dental College, Ghaziabad, Uttar Pradesh; 2 MDS, Professor and Head, Department of Pediatric and Preventive Dentistry, Shree Bankey Bihari Dental College, Ghaziabad, Uttar Pradesh; 3 MDS, Associate Professor, Department of Pediatric and Preventive Dentistry, Shree Bankey Bihari Dental College, Ghaziabad, Uttar Pradesh; 4 MDS, Senior Lecturer, Department of Pediatric and Preventive Dentistry, Shree Bankey Bihari Dental College, Ghaziabad, Uttar Pradesh; 5 MDS Student, Shree Bankey Bihari Dental College and Research Centre Ghaizabad, Uttar Pradesh

## Abstract

**Background:**

Dental anxiety is a frequent reason for reluctance in young children, leading to challenges in delivering effective dental treatment due to their uncooperative behavior. Aim: The aim of this study was to evaluate and compare the efficacy of different behaviour management techniques while administrating injectable LA with minimum pain perception and anxiety in children.

**Material and Methods:**

One hundred and twenty children, aged 4 to 10 years, were equally and randomly assigned to four groups. In Group I, local anesthesia was administered while using wireless headphones. For Group II, local anesthesia was administered using a mobile phone with earpieces. Group III received local anesthesia while incorporating the 3D virtual reality eyewear method and Group IV received local anesthesia while utilizing the stress ball technique. Pain and anxiety assessments were conducted using various scales and Physiological parameters such as pulse rate and oxygen saturation levels were recorded and the resultant data were systematically tabulated for subsequent statistical analysis.

**Results:**

Virtual reality group showed highly significant result in terms of lowering anxiety and pain scores compared to audio, audio visual, stress ball groups (*p*<0.001). Virtual group (5.10) also displays significantly highest behavior scores than the audio (1.70), audio visual group (3.96) and Stress Ball groups (2.66).

**Conclusions:**

The virtual reality group emerged as the most effective method in alleviating anxiety and pain experienced by pediatric dental patients.

** Key words:**Pain management, Anxiety, Distraction, Virtual reality device, Stress ball, Audiovisual, Audio.

## Introduction

As McElory (1895) wrote “Although operative dentistry may be perfect, the appointment is a failure if a child departs in tears.” In literature, for the first time, emphasis was placed on the effective management of a child’s behavior over technical expertise (1). Pediatric patients often respond in unpredicTable ways to dental treatment they may either accept dental treatment or maybe extremely fearful, stubborn resistant or reluctant to any kind of procedure. Pediatric dentist plays a vital responsibility in not just addressing the reported ailment in an anxious child but also in guiding the child on effective strategies to manage anxiety (2). Discomfort is an undesirable sensory and emotional condition regarded as the ‘fifth vital sign’ which needs to be carefully monitored especially while caring for paediatric patients (3). Anxiety is the most widespread of all human emotional states. It encompasses physical and mental sensations of powerlessness, the anticipation of an imminent threat, a sense of impending doom and danger arising from internal cognitive assessment, and an unresolved uncertainty regarding the nature of the threat and the most effective means to alleviate it (4). Hence, effective management of anxiety in children becomes a critical factor in ensuring successful dental care. Behavioral guidance strategies are employed to ease anxiety, foster a positive dental attitude, and facilitate the safe and efficient delivery of high-quality oral health care (5). Management approaches were proposed for alleviating children’s anxiety during dental treatment, categorized broadly into two modules. The initial segment comprises of behavioral management techniques such as the TSD technique, distraction, inspiration, modeling, and hypnotism. Additional segment includes pharmacological methods, with distraction being a particularly effective approach in reducing anxiety levels in children (6). Distraction is a frequently employed strategy in dental practice, redirecting a child’s focus away from potentially unpleasant procedures to captivating and fascinating stimuli. This approach is not only deemed safe and cost-effective but also contributes to creating a relaxed and effective experience, particularly during brief and mildly uncomforTable dental procedures (7). There are two distraction techniques commonly utilized in dentistry: auditory and audiovisual diversion. Auditory distraction encompasses music, audio presentations through headphones, and story telling, while AVD involves presenting stories on television, virtual reality, and three-dimensional video glasses (8). VR technology was initially acknowledged primarily for its entertainment value; nevertheless, over the last decade, its usage has been broadened to encompass a range of clinical fields, such as management of pain and the psychiatric disorders treatment. VR employs advance innovations to construct virtual environments (VEs) that submerge individuals in a virtual world. A benefit of utilizing VR methods in contrast to traditional behavioral management distraction is that it shields the patient from potentially stressful surroundings, replacing the view with personally selected relaxing content (9). An alternative approach to distraction involves utilizing stress balls, which could be a simpler yet cost-effective method for redirecting cognitive focus (10). The use of distraction techniques is extensively documented in medical settings for adults and is increasingly gaining popularity for application in children to mitigate dental anxiety. However, well-designed studies in this domain are still limited, and there are also some controversies surrounding the efficacy of distraction during dental treatment procedures (5).

AIM

The aim of this study is to compare the efficacy of different behavior management techniques while administrating injectable LA in order to investigate the minimum pain perception and anxiety in children. The objectives of the study were: 1) To determine the effectiveness of active distraction techniques as opposed to passive distraction techniques. 2) To determine the effect of distraction techniques on pain and anxiety levels in children receiving local anesthesia.

Inclusion criteria

a) Children aged 4 to 10 years.

b) Healthy subjects with no history of systemic disease.

c) Dental treatment requiring injectable local anesthetic.

d) Subjects exhibiting negative behaviour.

Exclusion criteria

a) Children below the age of 4 and above the age of 10 years.

b) History of systemic disease.

c) Dental treatment not requiring injectable LA.

d) Subjects exhibiting positive behaviour.

## Material and Methods

The ethical clearance was provided by institutional ethical committee vide reference number: (SBBDC/2022/383).

Pre- treatment, the dental fear and anxiety levels of all children were assessed 15 minutes before and 1 minute after needle removal from the tissue. Local anesthesia was administered using a standardized protocol (McDonald *et al*., 2011) by a single investigator. The intervention methods varied among 4 groups with 30 samples each:

Group I: Children were given the opportunity to choose their preferred story or music from an extensive collection of songs in the local regional language. They played their selected content through headphones during the local anesthesia administration. Group II: Children were inquired about their cartoon preferences, and the selected cartoon was shown to them using audio-visual (2D) aids through a mobile phone with earphones during the local anesthesia administration. Group III: Children were inquired about their cartoon preferences, and the selected cartoon was presented to them using VR glasses (3D) while local anesthesia administration. Group IV: Children were instructed to alternately squeeze and loosen the stress ball while the local anesthesia solution was being injected.

Physiological Parameters: The clinician conducting the study utilized a pulse oximeter to measure the individuals’ PR and SPO2 levels in order to assess their anxiety levels and perception of pain.

Non-physiological Parameters

1. Modified child dental anxiety scale (MCDAS) (11)

2. Wong- baker’s facial pain scale (12)

3. Venham’s anxiety rating scale (13)

4. Houpt behaviour rating scale

The data for the present study was entered in the Microsoft Excel and analyzed using the SPSS statistical software 23.0 Version.

## Results

In the present study based on total sample of 120, 55.0% were female and 45.0% were male. There was an unequal distribution of male and female in the study sample with higher representation of female as compared to male. The samples were distributed between the age of 4 years to 10 years. The mean age of the children in the Audio Group was 6.10 years, in the audio visual group was 7.13 years, in the Virtual Group was 7.03 and in the Stress Ball group was 6.86 years. No statistically significant difference was seen in distribution of study subjects by age (*p* > 0.005). Inter-group comparison of pulse rate from pre to post treatment levels was done using the One Way ANOVA ( Table 1). The intragroup change in pulse rate from pre to post treatment levels was statistically significant in all the four groups with decrease in pulse rate post operatively in the virtual, audio visual and stress ball groups while audio group was ineffective to decrease in pulse rate post operatively.(p<0.05).

**Figure 1:**
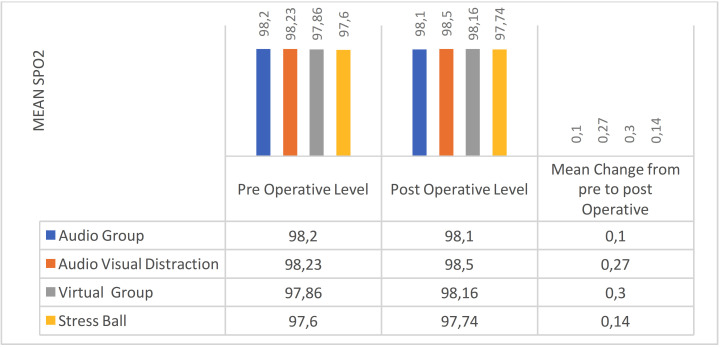
Inter-group comparison of SPO2 levels.

The intergroup comparison of mean change in SPO2 from pre to post treatment levels was done using the One Way ANOVA revealed insignificant difference between the groups (Fig. 1). The intragroup change in SPO2 from pre to post treatment levels was done using the One Way ANOVA. There was statistically non- significant difference in all the four groups with slight increase in SPO2 post operatively in the virtual, audio visual and stress ball group (*p*>0.05).

The intergroup comparison of mean anxiety scores assessed on the basis of MCDAS in the study subjects in four groups. Since the anxiety level based on the MCDAS were assessed at the pre-treatment level there was no difference in the anxiety levels which accepts our null hypothesis. There was non-significant variation in the dental anxiety scores between the four groups when the analysis was done using the One Way ANOVA ( Table 2).

**Figure 2:**
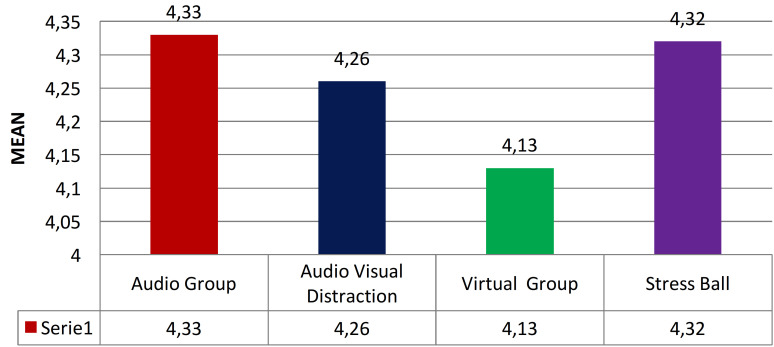
Inter-group comparison of Wong Baker’s Pain Rating Scale.

The intergroup comparison of mean pain scores assessed on the basis of WBPRS in the study subjects in four groups indicates a very highly significant difference between the groups (Fig. 2).

The intergroup comparison of dental anxiety assessed at the post treatment levels in the study participants in all four intervention groups using the VARS gave the *p* value of 0.001 which indicates a highly significant difference between the four groups with highest anxiety scores in the audio group and least in the virtual group ( Table 3).

**Figure 3:**
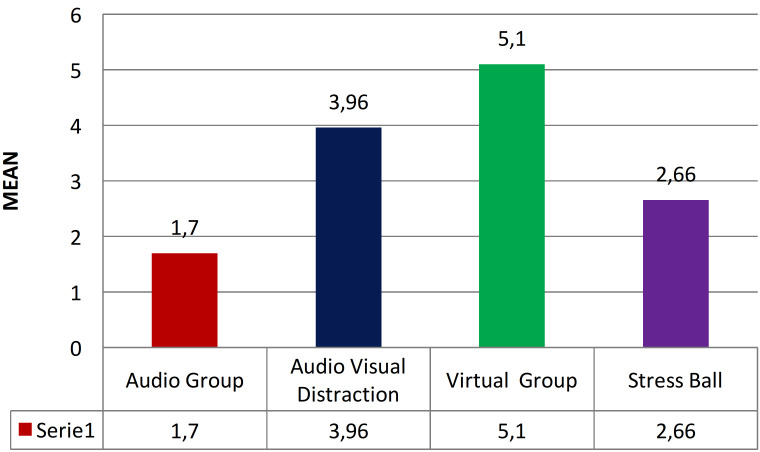
Inter-group comparison of Houpt Behaviour Rating Scale.

The intergroup comparison of child behavior on the HBRS which represents the amount of dental treatment meted out by the dentist during an appointment depending on the behavior of the child depicted a statistically significant result when analyzed using One Way One ANOVA with *p* value of less than 0.001(Fig. 3).

## Discussion

The primary focus of the pediatric dentist is to attain the child’s cooperation during different pediatric procedures in the dental clinic. The primary fear children experience during pediatric procedures is the painful administration of LA injections (13). Simply seeing needles and syringes can cause psychological trauma to the child, thus hindering the management of the child’s behavior. By alleviating the fear of pain associated with local anesthetic injections, children gain trust in the dentist, leading to better cooperation during treatment (12). The findings emphasize the effectiveness of VR eyeglasses in reducing anxiety during dental procedures for children aged 4 to 10 years. In this age range, children frequently exhibit negative behaviors during dental procedures (14).

In the present study, the PR was measured utilizing pulse oximeter at two time points - before and after the intervention. The findings indicated a notable difference between the groups, with the highest mean reduction observed in the Virtual Reality Group, followed by the Audio-Visual Distraction group, and the least reduction in the Stress Ball Group. However, in the Audio Group, there was an increase in the pulse rate from the pre to post-treatment time interval. The results are consistent with the study conducted by Halabi M *et al*. which also noted a significant difference in pulse rate scale between the three groups (control, AV eyeglasses ‘VR Box’, and wireless headphones) (9). The post-treatment survey showed that children preferred the VRD eyeglasses, reporting increased comfort, satisfaction, and decreased perception of pain and anxiety (15). This study also assessed the partial pressure of oxygen in reaction to different behavioral modification therapies within the four groups. The study by Koticha *P* *et al*. observed noTable difference in SpO2 before and after the extraction procedure in two groups when VR eyeglasses were utilized (6).

In the current study, pain scores were also assessed using the WBPRS in the study subjects. The study by Shetty V *et al*. also supports these outcomes, indicating a substantial decrease in pain perception among children using VR distraction (16). Significant reductions in dental pain were noted by Buldur B *et al*. in the groups utilizing distraction techniques (14). The study also presented the average anxiety scores based on the MCDAS, with no noTable differences noted among the four groups. Here, the post-treatment dental anxiety levels among the study participants in the various groups were evaluated using the VARS. A highly significant difference was observed among the four groups, with the highest anxiety scores recorded in the Audio Group and the lowest in the Virtual Group. The results are consistent with the study by Shetty V *et al*. showing a substantial decrease in anxiety levels among children using VR distraction with a marked reduction in salivary cortisol levels in children employing VR distraction (16). The study also outlines the mean scores from the HBRS with the highest mean score found in the Virtual Reality Group, followed by the Audio-Visual Group, the Stress Ball Group, and the lowest in the Audio Group. The statistically notable difference in mean scores among the four groups corresponds with the results of Alves IBS *et al*., where children felt more comforTable and satisfied during dental care when utilizing VR eyeglasses compared to other methods (15).

However, the use of AVD through a Tablet device fixed to the dental chair proved more effective in managing child behavior and controlling pain during IANB compared to employing VR Box and the control group. Notably, watching cartoons on TV was observed to have no impact on distracting children during anesthesia or alleviating their pain. VR distractions are safe, clinically practical, and require minimal pre-training from the practitioner; however, there are still certain limitations. Specifically, VR devices was initially created for adults and making it less suitable for children with smaller faces. Additionally, cost-effectiveness should be a crucial aspect of the dental anxiety management armamentarium, as the VRD devices utilized in this study are costly. These concerns need to be considered in future research.

## Conclusions

The Virtual reality group showed lowest pain score and the audio group showed highest pain score. The Virtual reality group showed highest behavior score and the audio group showed lowest behavior score. The intergroup and intragroup comparison of mean change in pulse rate from pre to post treatment levels revealed noTable difference between the groups with highest mean reduction in the VR group. The intergroup and intragroup comparison of mean change in SPO2 from pre to post treatment levels revealed insignificant difference between the groups. Therefore, it can be inferred that using VR eyewear as a distraction technique is effective in alleviating anxiety and pain in children, ultimately enhancing patient comfort in pediatric dental settings.

Clinical Significance.

The use of VR device can provide a marked reduction in fear and anxiety in pediatric patients which would allow the dental surgeon to provide a better oral health care to their patients.

## Figures and Tables

**Table 1:** Inter-group comparison of pulse rate.

	Pre Operative Level	Post Operative Level	Mean Change from pre to post Op	F value	P value	Significance
Audio Group	113.70±3.76	116.53±4.43	2.83±2.71	34.567	0.001	Significant
Audio Visual Distraction	112.47±3.90	100.43±0.54	-12.04±2.12			
Virtual Group	108.52±7.48	93.52±4.48	-15.00±2.16			
Stress Ball	110.43±6.32	106.30±5.37	-4.13±3.38			

**Table 2:** Inter-group comparison of Modified Child Dental Anxiety Scale.

	Mean	SD	Std Error	Minimum	Maximum	F value	P value
Audio Group	7.13	1.008	0.184	6.00	8.00	168.34	0.001 (Sig)
Audio Visual Distraction	2.93	1.014	0.185	2.00	4.00		
Virtual Group	1.31	1.105	0.205	.00	4.00		
Stress Ball	4.26	0.868	0.158	2.00	6.00		

**Table 3:** Inter-group comparison of Venham’s Anxiety Rating Scale.

	Mean	SD	Std Error	Minimum	Maximum	F value	P value
Audio Group	3.70	0.749	0.136	2.00	5.00	106.86	0.001 (Sig)
Audio Visual Distraction	1.36	0.490	0.089	2.00	3.00		
Virtual Group	0.82	0.468	0.086	.00	2.00		
Stress Ball	1.96	0.413	0.075	1.00	3.00		

## Data Availability

The datasets used and/or analyzed during the current study are available from the corresponding author.
